# College Sociability

**Published:** 1880-04

**Authors:** 


College Sociability.—One of the most pleasant social occasions enjoyed by students during the present college year, was the union of the senior classes of the several departments or colleges of the Northwestern University, better known as the Chicago Medical College, the Union College of Law, and the College of Liberal Arts, at the residence of Dr. and Mrs. N. S. Davis, in Evanston, on the afternoon of the 13th March, last. After partaking of a bountiful lunch and enjoying an hour or two of free social intercourse, the company repaired to the University campus, examined the different buildings, including the Woman's College and the Theological school, and were received at the University Hall by President Marcy, aided by Professor Carhart, shown through the museum and into the library, where they tarried long enough to enjoy some impromptu sentiments and responses from representatives of the three classes. At the Woman's College the party enjoyed some excellent music, after which the law and medical classes returned to the city, highly pleased with their afternoon's entertainment.

Medical College Commencements.—We give in this number an account of the commencement exercises of the Rush Medical College. Those of the Medical Department of the Northwestern University, better known as the Chicago Medical College, do not occur until March 30th, and consequently will not be noticed until our next issue.

